# Dietary Protein Intake and Single-Nephron Glomerular Filtration Rate

**DOI:** 10.3390/nu12092549

**Published:** 2020-08-23

**Authors:** Rina Oba, Go Kanzaki, Takaya Sasaki, Yusuke Okabayashi, Kotaro Haruhara, Kentaro Koike, Akimitsu Kobayashi, Izumi Yamamoto, Nobuo Tsuboi, Takashi Yokoo

**Affiliations:** Division of Nephrology and Hypertension, Department of Internal Medicine, The Jikei University School of Medicine, 3-25-8 Nishi-Shimbashi, Minato-ku, Tokyo 105-8461, Japan; h21ms-oba@jikei.ac.jp (R.O.); h18ms-sasaki@jikei.ac.jp (T.S.); kmckr843@yahoo.co.jp (Y.O.); Kotaro.Haruhara@monash.edu (K.H.); kkoike@jikei.ac.jp (K.K.); akimitsu@kk.iij4u.or.jp (A.K.); izumi26@jikei.ac.jp (I.Y.); tsuboi-n@jikei.ac.jp (N.T.); tyokoo@jikei.ac.jp (T.Y.)

**Keywords:** glomerular hyperfiltration, nephron number, protein intake, single-nephron glomerular filtration rate

## Abstract

High protein intake can increase glomerular filtration rate (GFR) in response to excretory overload, which may exacerbate the progression of kidney disease. However, the direct association between glomerular hemodynamic response at the single-nephron level and dietary protein intake has not been fully elucidated in humans. In the present study, we evaluated nutritional indices associated with single-nephron GFR (SNGFR) calculated based on corrected creatinine clearance (SNGFR_Cr_). We retrospectively identified 43 living kidney donors who underwent enhanced computed tomography and kidney biopsy at the time of donation at Jikei University Hospital in Tokyo from 2007 to 2018. Total nephron number was estimated with imaging-derived cortical volume and morphometry-derived glomerular density. SNGFR_Cr_ was calculated by dividing the corrected creatinine clearance by the number of non-sclerosed glomeruli (Nglom_NSG_). The mean (± standard deviation) Nglom_NSG_/kidney and SNGFR_Cr_ were 685,000 ± 242,000 and 61.0 ± 23.9 nL/min, respectively. SNGFR_Cr_ was directly associated with estimated protein intake/ideal body weight (*p* = 0.005) but not with body mass index, mean arterial pressure, albumin, or sodium intake. These findings indicate that greater protein intake may increase SNGFR and lead to glomerular hyperfiltration.

## 1. Introduction

Chronic kidney disease (CKD) is a global health concern because of increased morbidity and mortality [1]. Dietary interventions are an effective strategy to prevent or delay the progression of CKD, and current nutritional management recommendations include controlling the intake of protein, sodium, and phosphorus [2]. A high-protein diet is known to increase the risk of CKD development and progression through several mechanisms. In animal models [3,4] and humans [5,6], dietary protein was shown to increase intrinsic acid production, which can cause kidney injury due to acid retention-induced kidney endothelin and aldosterone production. Furthermore, increased dietary protein per se was shown to induce kidney injury through a hemodynamic mechanism of renal hyperfiltration in humans [7] and experimental animals [8]. However, the direct association between protein intake and glomerular hemodynamic response remains poorly understood.

Glomerular hyperfiltration, defined as an abnormal renal hemodynamic change at a single-nephron or whole-kidney level, may arise from metabolic disturbances and is a risk factor for progressive kidney damage. Among the various diseases and conditions associated with glomerular hyperfiltration are diabetes mellitus, polycystic kidney disease, secondary focal segmental glomerulosclerosis, pregnancy, obesity, and a high-protein diet [9]. While experimental studies using high protein loading in animals show that glomerular hyperfiltration at the single-nephron level precedes the subsequent albuminuria and glomerulosclerosis [10], the lack of available methods to measure single-nephron glomerular filtration rate (SNGFR) in humans has hindered clinical studies to assess glomerular hyperfiltration in humans [11]. Thus, most studies have utilized elevated glomerular filtration rate (GFR) at the whole-kidney level as an indicator of glomerular hyperfiltration.

Recent studies in humans have described a new method to calculate SNGFR, which is defined as GFR divided by the estimated total number of non-sclerosed glomeruli (Nglom_NSG_) [12,13]. Using this method, we previously demonstrated that the estimated total number of glomeruli (Nglom_TOTAL_) in Japanese living kidney donors was similar to that determined in a Japanese autopsy study which used the physical disector/fractionator method, the gold standard method for estimating total number of glomeruli in kidneys [14,15,16]. Additionally, in an autopsy study of Japanese subjects, we showed that SNGFR was higher in those with hypertension compared to normotensive subjects [14]. A higher SNGFR, which indicates glomerular hyperfiltration, is associated with certain risk factors for CKD and certain kidney biopsy findings such as glomerular hypertrophy and sclerosis [13,14]. Despite numerous clinical trials and observational nutritional studies on renal hyperfiltration using elevated whole-kidney GFR as a parameter, few studies reported the association of SNGFR with nutritional indices.

We hypothesized that a high protein intake would lead to glomerular hyperfiltration with an elevated SNGFR, leading to CKD either directly or indirectly through damage to the glomerular structure. In the present study, we calculated SNGFR by estimating the number of glomeruli in living kidney donors using the combined computed tomography imaging and kidney biopsy method [15] and explored the association between protein intake and SNGFR in healthy individuals without CKD.

## 2. Materials and Methods

### 2.1. Subjects

This retrospective cohort study included donors who underwent enhanced computed tomography imaging and kidney biopsies at the time of donation at Jikei University Hospital in Tokyo, Japan, between 1 January 2007 and 31 December 2018. All kidney donors were selected according to the Amsterdam Forum guidelines [17]. None of the subjects had a history of diabetes based on a National Glycohemoglobin Standardization Program-standardized glycated hemoglobin level of <6.2%. A history of hypertension was not an exclusion criterion in subjects with blood pressure within normal limits (systolic blood pressure <130 mmHg, diastolic blood pressure <80 mmHg) controlled with diet therapy or medication. Kidney biopsy specimens with <2 mm^2^ cortex and/or <4 non-sclerotic glomeruli were excluded based on the findings of previous studies [12,15]. Subjects with missing data on 24-h urine collection were also excluded.

This study was approved by the ethics review board of Jikei University School of Medicine (30-060, 9081).

### 2.2. Measurements

General demographic data, including age, height, body weight, medical history, treatment, and blood pressure, were obtained from the medical records before donation. Laboratory measurements included urea nitrogen, creatinine, estimated GFR (eGFR), total protein, albumin, uric acid, and inorganic phosphate. Urine was collected for 24 h.

Mean arterial pressure (MAP) was defined as diastolic blood pressure plus pulse pressure divided by 3. Body mass index (BMI) was defined as body weight divided by height squared. Body surface area was calculated from the Dubois formula as follows: body surface area (m^2^) = body weight^0.425^ × height^0.725^ × 0.007184 [18]. Creatinine clearance was determined using the standard clearance technique based on 24-h urine collection. eGFR was calculated using the following formula for Japanese subjects: eGFR (mL/min/1.73 m^2^) = 194 × creatinine^−1.094^ × age^−0.287^ (×0.7399 for females) [19]. Effective renal plasma flow was calculated from ^99m^Tc-MAG3 clearance values using a previously validated equation: effective renal plasma flow (mL/min) = 1.86 × ^99m^Tc-MAG3 clearance + 4.6 [20].

### 2.3. Data on Dietary Intake

All subjects were on an unrestricted protein diet before donation. A 24-h urine collection was obtained before the kidney transplantation. Dietary sodium intake (SI) was assessed by 24-h urinary sodium excretion. Dietary protein intake was estimated from 24-h urinary urea excretion according to Maroni’s formula [21], and protein intake (PI) was normalized to ideal body weight (IBW) and expressed as g/kg/day (PI/IBW). IBW was defined as a BMI of 22 kg/m^2^, according to the 2016 Japan Society for the Study of Obesity guidelines, as reported previously [22].

### 2.4. Estimation of Single-Nephron Glomerular Filtration Rate

The Nglom_TOTAL_ was estimated based on a previously reported method [12,15]. Briefly, a two-step approach was used to calculate SNGFR based on corrected creatinine clearance (SNGFR_Cr_). First, we multiplied the glomerular density of kidney biopsy specimens collected at the time of kidney donation by the total renal cortical volume obtained from enhanced computed tomography images. Cortical and whole kidney volumes were measured using the ITK-SNAP software (version 1.1, University of Pennsylvania, Philadelphia, PA, USA), as previously described [15]. The total number of glomeruli was divided by 2 to determine the number per kidney, by 1.43 to account for the decrease in tissue volume due to paraffin embedding, and by 1.268 to account for the decrease in volume due to loss of tissue perfusion pressure. The degree of non-sclerotic and sclerotic glomerular profiles was calculated for each kidney specimen and then multiplied by the Nglom_TOTAL_ to obtain the numbers of non-sclerotic glomeruli (Nglom_NSG_) and globally sclerotic glomeruli (Nglom_GSG_). Second, we calculated SNGFR_Cr_ as the corrected creatinine clearance divided by the Nglom_NSG_. In the present study, a correlation coefficient of 0.715 was used to eliminate the overestimation of GFR based on creatinine clearance according to the previous study as follows: SNGFR_Cr_ = 24-h creatinine clearance × 0.715/(Nglom_NSG_ × 2) [19].

Mean glomerular volume (Vglom) was estimated from the measured mean glomerular tuft area (GA) as follows: Vglom = (GA)^3/2^ × β/d, where β is a dimensionless shape coefficient (β = 1.382 for spheres) and d is the size distribution coefficient used to adjust for variations in glomerular size (d = 1.01) [23].

### 2.5. Statistical Analysis

We first described baseline characteristics in the overall cohort of 43 subjects as well as in the low, intermediate, and high PI/IBW groups according to the PI/IBW tertiles. Continuous variables were presented as means ± standard deviation or numbers with percentages. The Jonckheere–Terpstra or the Mantel–Haenszel test for trend was used to analyze trends in baseline characteristics and morphological measurements among the PI/IBW groups. Pearson’s correlation coefficient was used to assess correlations between two variables. Multiple regression analysis was used to evaluate the association of SNGFR_Cr_ with PI/IBW, adding potential confounders [9,13] as covariates and testing for the presence of interactions among the factors. Statistical significance was defined as a *p* < 0.05. All statistical analysis was performed using SPSS v.25.0 (IBM Corp., Armonk, NY, USA) and GraphPad PRISM 8.0 (GraphPad Software, San Diego, CA, USA).

## 3. Results

### 3.1. Subjects Characteristics

Table 1 lists the demographic, clinical, and laboratory data of 43 donors included in the study. The mean age was 56.4 ± 10.2 years (mean ± standard deviation); 35% of the subjects were male, and the mean eGFR was 76.9 ± 12.9 mL/min/1.73 m^2^. The BMI was 23.3 ± 3.0 kg/m^2^, which was almost within the normal BMI range of 18.5–24.9 kg/m^2^ based on the Japan Society for the Study of Obesity [24]. There were 7 (16%) subjects with mild hypertension among the 43 donors. The demographic and biochemical characteristics, including age, BMI, blood pressure, renal function, and nutritional indices, were almost comparable among the groups. The SI tended to increase in correlation with the PI/IBW.

### 3.2. Histopathological Characteristics and Morphometric Data

A summary of the renal histopathological characteristics and morphometric data of the study cohort are presented in Table 2. Briefly, there were 733,000 ± 237,000 Nglom_TOTAL_/kidney and 685,000 ± 242,000 Nglom_NSG_/kidney. There were no significant differences in Nglom_TOTAL_, Nglom_NSG_, Nglom_GSG_, Vglom, kidney volume, cortical volume, degree of glomerular sclerosis, and interstitial fibrosis and/or tubular atrophy among the three groups.

### 3.3. Diet-Related Parameters according to Single-Nephron and Whole-Kidney GFR

**Figure 1:**
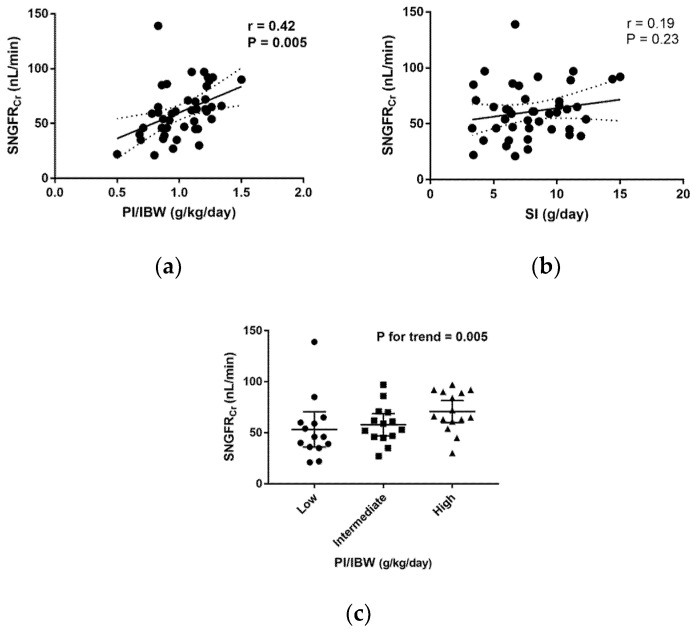
Correlations between diet-related parameters and single-nephron glomerular filtration rate. Associations (**a**) between estimated protein intake/ideal body weight (PI/IBW) and single-nephron glomerular filtration rate (SNGFR_Cr_), and (**b**) between sodium intake (SI) and SNGFR_Cr_. Measures of association were tested by Pearson’s correlation coefficient. Solid lines indicate lines of best fit. Dotted lines show 95% confidence intervals. (**c**) SNGFR_Cr_ values in the low, intermediate, and high PI/IBW groups. Bold lines indicate 95% confidence intervals. The statistical test for trend shows that SNGFR_Cr_ tends to increase significantly with increasing PI/IBW. Differences among groups are analyzed by the Jonckheere–Terpstra test.

The mean SNGFR_Cr_ was 61.0 ± 23.9 nL/min. Our analysis revealed that PI/IBW exhibited a positive linear correlation with SNGFR_Cr_ (*r* = 0.42, *p* = 0.005), whereas SI did not show a significant correlation with SNGFR_Cr_ (*r* = 0.19, *p* = 0.23) (Figure 1a,b). The trend analysis revealed similar results: SNGFR_Cr_ increased significantly with increasing PI/IBW (*p* for trend = 0.005) (Figure 1c). Furthermore, the evaluation of the single-nephron parameter Vglom and the whole-kidney parameters eGFR, kidney volume, and cortical volume for their correlation with PI/IBW and SI (Table 3) revealed that neither PI/IBW nor SI showed a statistically significant correlation with eGFR, Vglom, kidney volume, and cortical volume.

### 3.4. Dietary Factors and SNGFR_Cr_

To confirm the significant and independent association between PI/IBW and SNGFR_Cr_, we further performed multiple linear regression analyses for SNGFR_Cr_, including PI/IBW, height, BMI, MAP, albumin, and SI. The association of SNGFR_Cr_ with PI/IBW remained significant (*p* < 0.05) after adjustment for all the identified confounders of glomerular hyperfiltration (Table 4).

## 4. Discussion

The major new finding of the present study, including 43 Japanese living kidney donors without CKD, is the positive correlation between protein intake and SNGFR, which persisted with the statistical trend analysis of the cohort categorized according to the PI/IBW tertiles and after the consideration of confounders for glomerular and renal hyperfiltration. These findings suggest that greater daily protein intake may increase SNGFR and induce glomerular hyperfiltration.

The positive correlation of protein intake with SNGFR is in agreement with previous reports. Early studies in rats demonstrated that an increase in SNGFR depended on protein intake during systemic amino acid infusion [25,26]. Although the exact mechanism for the glomerular hemodynamic responses to a high-protein diet, as well as the multiple mediators and factors affecting this mechanism, is yet to be settled, several studies reported that neuronal nitric oxide synthase [27,28] and tubuloglomerular feedback (TGF) [29,30] played pivotal roles in high-protein diet-induced glomerular hyperfiltration [31]. Specifically, TGF was a key player in the control of glomerular hemodynamics. Micropuncture data showed that a high-protein diet induced a 21% increase in SNGFR with TGF but no significant change in SNGFR in the absence of TGF [8].

High protein intake increases the filtration of amino acids and their reabsorption in proximal tubules. As the reabsorption of most amino acids is sodium-dependent [32], concomitantly increased sodium reabsorption leads to reduced sodium chloride concentrations in macula densa and results in reduced TGF signaling, which increases the SNGFR [33,34]. High protein intake also leads to increased urea excretion, which creates an osmotic load in the renal tubule lumen. As the most abundant solute in urine, urea plays an important role in the urinary concentrating mechanism [35]. Vasopressin, which is secreted to prevent urea-dependent osmotic diuresis, affects urea channels, resulting in the reabsorption of urea into the inner medullary interstitium. Sodium reabsorption that occurs simultaneously to create and maintain the osmotic gradient in the medulla [35,36] leads to increased SNGFR, as mentioned above.

Although not observed in this study, an association between high protein intake and elevated eGFR has been reported in several studies [7,37]. This discrepancy might be caused by a difference in glomerular hyperfiltration between the single-nephron level and the whole-kidney level [5]. In addition, in the early stage of CKD, whole-kidney GFR is preserved within the normal range, whereas SNGFR increases to compensate for nephron loss [38]. Furthermore, in this study, the subjects were healthy kidney donors with preserved kidney function and a mean PI/IBW of 1.0 ± 0.2 g/kg/day, whereas other studies defined a high-protein diet as a PI/IBW of >1.2 or 1.5 g/kg/day [39,40]. According to the International Society of Renal Nutrition and Metabolism Commentary on the National Kidney Foundation and Academy of Nutrition and Dietetics KDOQI Clinical Practice Guideline for Nutrition in Chronic Kidney Disease, a moderately high protein diet was defined as 1.2–1.5 g/kg/day; this was the reported average protein intake of adults without CKD in the United States [41]. In this updated guideline, the recommended range for adults without CKD but who are at high risk for CKD was 0.8–1.0 g/kg/day. The optimal daily protein intake for healthy individuals is a topic of much debate.

Unlike a previous study that determined the SNGFR based on urinary iothalamate clearance in healthy adult subjects [13], we used creatinine clearance to calculate SNGFR as we were able to correct most of the data based on 24-h urine collection. Creatinine clearance is known to lead to the overestimation of GFR because of the tubular secretion of creatinine [42]. Applying a correlation coefficient [19] to calculate SNGFR based on creatinine clearance allowed us to address this issue in the present study.

Moreover, SI and MAP did not show a significant correlation with SNGFR_Cr_ in the present study. In fact, the direct association of SNGFR with SI and hypertension is not fully understood. Certain studies have shown that salt intake increased GFR at a whole-kidney level and mean arterial pressure [43,44]. A recent study calculating SNGFR in 1388 living kidney donors showed that a higher SNGFR was associated with higher BMI, increased height, and family history of end-stage renal disease, although it was not associated with mild hypertension [13]. In an autopsy study of Japanese patients, we previously reported that SNGFR was higher in hypertensive subjects than normotensive subjects [14]; however, the blood pressure of the subjects in the present study was well controlled with medications or dietary interventions. In addition, the mean SI was within the normal range (8.0 ± 2.9 g/day) compared to the mean SI of 10.1 g/day reported in the Japanese population. Of note, the recommended SI is less than 7.5 g/day for males and 6.5 g/day for females according to the 2020 recommendations of the Japanese Ministry of Health, Labor, and Welfare.

The present study has several limitations that should be acknowledged. First, parameters representing dietary acid load were not included. Second, we estimated SNGFR based on only creatinine clearance, and dietary protein intake was estimated from 24-h urinary urea excretion at one point. Third, the causal relationship between dietary protein intake and SNGFR could not be determined due to the correlation analyses and the observational nature of the study. Finally, the study cohort included Japanese subjects with normal kidney function, and the generalization of the results require further studies. Recently, in our latest study, we proposed a new method to estimate nephron number using unenhanced computed tomography and biopsy-based stereology [45]. Therefore, further study on a larger number of subjects, including CKD patients who are often unsuitable for contrast media administration, is expected with this new method.

Despite these limitations, a major strength of the present study is the measurement of SNGFR in humans as a representative parameter of renal hemodynamic change at the single-nephron level. Few studies previously investigated SNGFR in association with protein intake. We also examined the correlation of SNGFR with diet-related parameters to reveal the strong association between protein intake and glomerular hyperfiltration.

## 5. Conclusions

The present study, including healthy Japanese living kidney donors, reveals that greater protein intake might lead to increased SNGFR and glomerular hyperfiltration. These findings gave us a new insight to understand the critical role of a high-protein diet in inducing glomerular hyperfiltration.

## Figures and Tables

**Table 1:** Clinical characteristics of living kidney donors at the time of donation in the entire cohort and in groups according to the tertiles of protein intake per ideal body weight.

	All (*n* = 43)	Low PI/IBW (≤0.88 g/kg/day) (*n* = 14)	Intermediate PI/IBW (0.89–1.13 g/kg/day) (*n* = 14)	High PI/IBW (>1.13 g/kg/day) (*n* = 15)	*p* for Trend
Age, years	56.4 ± 10.2	51.6 ± 11.2	61.1 ± 8.4	56.3 ± 9.1	0.27
Male, *n* (%)	15 (35)	5 (36)	4 (29)	6 (40)	0.80
MAP (mmHg)	85.7 ± 9.5	85.8 ± 12.7	86.5 ± 6.5	85.0 ± 8.9	0.65
Hypertension, *n* (%)	7 (16)	3 (21)	2 (14)	2 (13)	0.56
Height (m)	1.61 ± 0.08	1.62 ± 0.10	1.59 ± 0.08	1.62 ± 0.08	0.79
BMI (kg/m^2^)	23.3 ± 3.0	23.8 ± 3.6	22.8 ± 2.5	23.2 ± 3.0	0.71
BSA (m^2^)	1.63 ± 0.16	1.66 ± 0.17	1.58 ± 0.15	1.65 ± 0.17	0.71
UN (mg/dL)	13.5 ± 3.0	12.3 ± 3.4	13.9 ± 3.1	14.3 ± 2.3	0.12
Cr (mg/dL)	0.70 ± 0.11	0.68 ± 0.10	0.70 ± 0.12	0.72 ± 0.13	0.56
eGFR (mL/min/1.73 m^2^)	76.9 ± 12.9	81.4 ± 13.0	73.6 ± 13.6	75.8 ± 11.6	0.21
ERPF (mL/min)	224 ± 62	209 ± 46	210 ± 48	250 ± 79	0.11
TP (g/dL)	7.1 ± 0.4	7.1 ± 0.4	7.1 ± 0.4	7.1 ± 0.4	0.36
Alb (g/dL)	4.2 ± 0.3	4.1 ± 0.3	4.3 ± 0.4	4.3 ± 0.2	0.13
UA (mg/dL)	4.9 ± 1.2	5.0 ± 1.1	4.3 ± 1.5	5.3 ± 0.9	0.26
Pi (mg/dL)	3.6 ± 0.4	3.7 ± 0.4	3.6 ± 0.4	3.6 ± 0.4	0.64
SI (g/day)	8.0 ± 2.9	6.7 ± 2.8	7.2 ± 2.1	10.0 ± 2.8	0.003

Data are presented as means ± standard deviation or numbers with percentages in parentheses. Abbreviations: Alb, albumin; BMI, body mass index; BSA, body surface area; Cr, serum creatinine; eGFR, estimated-glomerular filtration rate; ERPF, effective renal plasma flow; MAP, mean arterial pressure; Pi, inorganic phosphate; PI/IBW, estimated protein intake/ideal body weight; SI, sodium intake; TP, total protein; UA, uric acid; UN, urea nitrogen.

**Table 2:** Histopathological characteristics and morphometric data among all subjects and according to protein intake per ideal body weight tertiles.

	All (*n* = 43)	Low PI/IBW (≤0.88 g/kg/day) (*n* = 14)	Intermediate PI/IBW (0.89–1.13 g/kg/day) (*n* = 14)	High PI/IBW (>1.13 g/kg/day) (*n* = 15)	*p* for Trend
Nglom_TOTAL_ (/kidney)	733,000 ± 237,000	832,000 ± 296,000	666,000 ± 158,000	702,000 ± 222,000	0.23
Nglom_NSG_ (/kidney)	685,000 ± 242,000	767,000 ± 325,000	642,000 ± 183,000	649,000 ± 191,000	0.23
Nglom_GSG_ (/kidney)	48,000 ± 97,000	64,000 ± 120,000	25,000 ± 63,000	54,000 ± 100,000	0.95
Vglom (×10^6^ μm^3^)	2.39 ± 1.03	2.23 ± 1.03	2.69 ± 1.16	2.24 ± 0.89	0.93
Kidney volume (cm^3^/kidney)	125 ± 25.4	124 ± 29.7	122 ± 22.6	129 ± 24.9	0.66
Cortical volume (cm^3^/kidney)	89.5 ± 20.2	92.0 ± 27.3	86.6 ± 15.5	89.9 ± 17.1	0.77
GS (%)	5.64 ± 9.85	5.39 ± 9.95	5.35 ± 8.81	6.16 ± 11.2	1.00
IF/TA (%)	5.48 ± 5.93	6.43 ± 6.38	2.71 ± 2.52	7.17 ± 7.07	0.31

Data are presented as means ± standard deviation or numbers with percentages in parentheses. Abbreviations: GS, glomerular sclerosis; IF/TA, interstitial fibrosis and/or tubular atrophy; Nglom_GSG_, number of globally sclerosed glomeruli; Nglom_NSG_, number of non-sclerosed glomeruli; Nglom_TOTAL_, total nephron number; PI/IBW, estimated protein intake/ideal body weight; Vglom, mean glomerular volume.

**Table 3:** Correlation analyses of study parameters.

	eGFR (mL/min/1.73 m^2^)		Vglom (×10^6^ μm^3^)		Kidney Volume (cm^3^/Kidney)		Cortical Volume (cm^3^/Kidney)	
	*r* Coefficient	*p* Value	*r* Coefficient	*p* Value	*r* Coefficient	*p* Value	*r* Coefficient	*p* Value
PI/IBW (g/kg/day)	−0.11	0.48	0.03	0.87	0.14	0.38	−0.08	0.63
SI (g/day)	0.15	0.33	−0.15	0.33	0.15	0.34	0.05	0.75

Abbreviations: eGFR, estimated-glomerular filtration rate; PI/IBW, estimated protein intake/ideal body weight; SI, sodium intake; Vglom, mean glomerular volume.

**Table 4:** Factors associated with single-nephron glomerular filtration rate by multiple linear regression analysis.

	Univariable		Multivariable Model 1		Multivariable Model 2	
	SNGFR_Cr_ (nL/min)		SNGFR_Cr_ (nL/min)		SNGFR_Cr_ (nL/min)	
	β Coefficient	*p* Value	β Coefficient	*p* Value	β Coefficient	*p* Value
PI/IBW (g/kg/day)	0.42	0.005	0.43	0.004	0.49	0.01
Height (m)	0.08	0.62	0.08	0.60	0.15	0.37
BMI (kg/m^2^)	0.10	0.51	0.14	0.33	0.16	0.38
MAP (mmHg)	0.03	0.83	-	-	−0.04	0.80
Alb (mg/dL)	0.03	0.87	-	-	−0.10	0.55
SI (g/day)	0.19	0.23	-	-	−0.14	0.51

In model 2, one subject was excluded because of missing data on mean arterial pressure (MAP). Abbreviations: Alb, albumin; BMI, body mass index; MAP, mean arterial pressure; PI/IBW, estimated protein intake/ideal body weight; SI, sodium intake; SNGFR_Cr_, single-nephron glomerular filtration rate.
